# Future opportunities and nuances with the use of PSMA PET in prostate cancer (MD PET 1)

**DOI:** 10.7150/thno.132930

**Published:** 2026-04-09

**Authors:** Alicia K. Morgans, Christopher M. Pieczonka, Evan Y. W. Yu, Himanshu Nagar, Leonard G. Gomella, Medhat M. Osman, Phillip J. Koo, Steven E. Finkelstein

**Affiliations:** 1 Department of Medical Oncology, Dana-Farber Cancer Institute, Boston, MA, USA.; 2 U.S. Urology Partners and Associated Medical Professionals of New York, Syracuse, NY, USA.; 3 Fred Hutchinson Cancer Center and University of Washington, Seattle, WA, USA.; 4 Memorial Sloan Kettering Cancer Center, New York, NY, USA.; 5 Department of Urology, Thomas Jefferson University, Philadelphia, PA, USA.; 6 Department of Radiology, Division of Nuclear Medicine, Saint Louis University Hospital, St. Louis, MO, USA.; 7 Prostate Cancer Foundation, Santa Monica, CA, USA.; 8 Center of Advanced Radiation Excellence (CARE) and Radiation Oncology Research, Associated Medical Professionals / US Urology Partners, Syracuse, NY, USA.

**Keywords:** MD PET, prostate cancer, prostate-specific membrane antigen, PSMA-PET/CT, clinical decision-making, diagnostic imaging

## Abstract

Imaging with prostate-specific membrane antigen (PSMA) PET has significantly improved prostate cancer staging with superior diagnostic performance compared to conventional methods. Although it is increasingly adopted in clinical practice, several barriers hinder its full integration into routine workflows. This review highlights the existing knowledge gaps, infrastructure limitations, and inconsistencies in interpretation that affect the utility of PSMA PET across healthcare settings. We examine the potential reasons behind variability in scan performance, including scanner design, detector technology, sensitivity, and resolution, as well as the accreditation status of the facilities and reader expertise. We also highlight the inconsistent understanding of PSMA PET ordering practices, particularly among urologists, and the influence of ownership-driven utilization, both of which contribute to underuse and overuse. Radiology reporting that lacks sufficient detail and a shortage of trained nuclear medicine specialists may present additional challenges to effective treatment planning, although diagnostic radiologists also contribute to PET scan interpretation. This review highlights the potential role of standardized reporting protocols, accreditation, expanded education, and integration of AI tools in enhancing diagnostic accuracy and consistency. Additionally, we examine the impact of PSMA PET on clinical decision-making in unfavorable intermediate-, high risk-, and biochemically recurrent prostate cancer, as well as the emerging role of PSMA PET-derived metrics in staging, biopsy guidance, and treatment planning. While PSMA PET has shown value in modifying management strategies, its clinical benefit requires validation through future, prospective, outcome-driven studies. In addition, emerging applications of PSMA PET in non-prostate malignancies hold the potential to transform diagnostic and therapeutic approaches beyond prostate cancer.

## Introduction

Prostate cancer, the second most common cancer in men, accounts for approximately 7.3% of the global cancer burden 1. In the United States, it represents 15.4% of all new cancer diagnoses, with an estimated 313,830 new cases and 36,320 deaths projected for 2026 2. The 5-year relative survival rate is nearly 100% for localized and regional disease but declines to 37.9% for metastatic prostate cancer 3. From a therapeutic point of view, clinically important improvements in survival from addition of abiraterone to ADT have been reported to be maintained for longer than 7 years in very high risk localized prostate cancer either with positive nodes or 2 of 3 criteria being Gleason 8-10, PSA>40 ng/mL and/or T3-4 status. 4. Therefore, early and accurate staging is crucial for determining therapeutic strategies and reducing prostate cancer-specific mortality. Imaging plays a pivotal role from diagnosis and staging to the detection of recurrence and confirming eligibility of patients prior to prostate-specific membrane antigen (PSMA)-targeted radioligand therapy. There is also significant interest in integrating PSMA PET image-based information from radiomics into a multiomics framework, with the aim to combine biomolecular-level information with imaging data. The clinical application of radiomics can help to identify the relationship between imaging results and the clinical outcome of interest 5.

Historically, conventional imaging modalities such as computed tomography (CT) and magnetic resonance imaging (MRI) have had limitations in detecting small lymph node metastases, distant metastases, as well as early recurrence 6-8. Bone scintigraphy, although commonly used to detect bone metastases, has a high false-positive rate, which can lead to overstaging of disease 8. These diagnostic pitfalls underscored the need for more sensitive and specific imaging techniques, which can detect previously occult disease with greater accuracy and at the earlier stages of disease progression. The emergence of PSMA positron emission tomography (PET) has helped address many of these challenges 9-11. Notably, it enables the detection of PSMA-positive lesions in patients with negative conventional imaging, underscoring the diagnostic value of PSMA-targeted molecular imaging 12.

**Figure 1:**
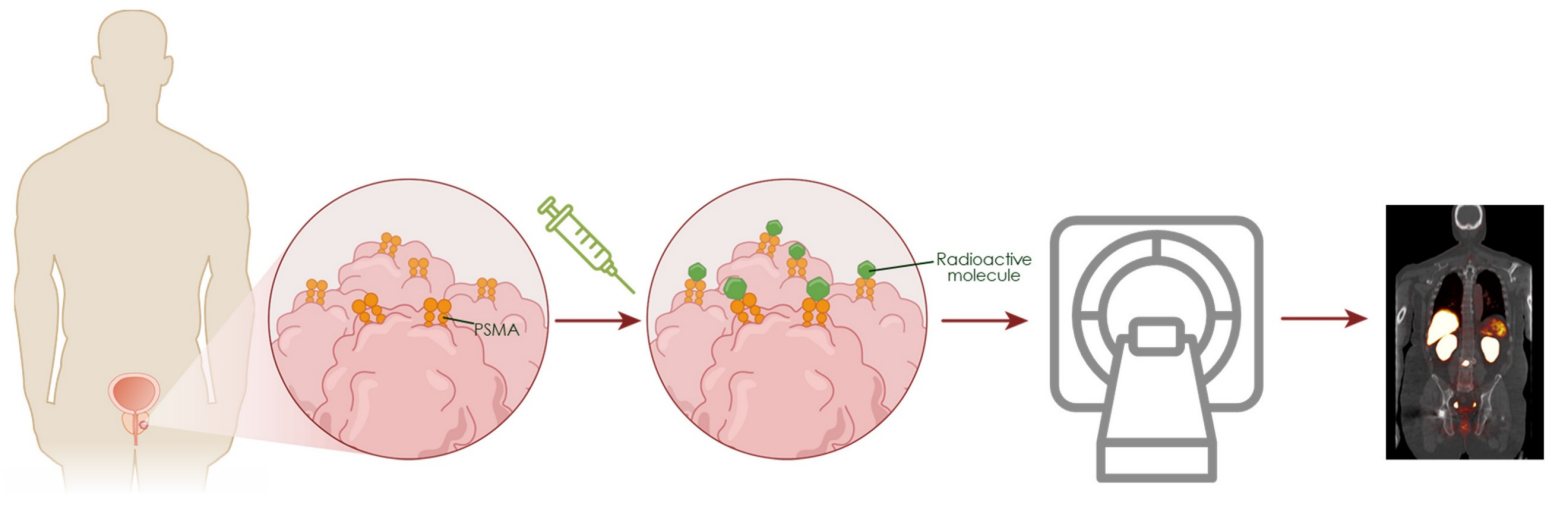
** Imaging with PSMA PET.** PSMA is a transmembrane glycoprotein overexpressed on the surface of prostate cancer cells compared to normal tissues. When PSMA PET radiotracers are injected into the bloodstream they bind PSMA, allowing for specific detection of prostate tumors/metastases through PET.

PSMA is a transmembrane glycoprotein markedly overexpressed in prostate cancer cells compared to normal tissues, making it an ideal target for molecular imaging 13,14. PSMA PET tracers radiolabeled with isotopes such as gallium-68 (^68^Ga) or fluorine-18 (^18^F), bind specifically to the extracellular domain of PSMA and undergo internalization via endocytosis. This accumulation enables high-contrast imaging of PSMA-expressing lesions (Figure 1). Currently, the U.S. Food and Drug Administration (FDA)-approved PSMA PET agents include piflufolastat F 18 (PYLARIFY, PYLARIFY TRUVU), flotufolastat F 18 (POSLUMA), and ^68^Ga-PSMA-11 (LOCAMETZ, ILLUCCIX, GOZELLIX) 15-20. All are indicated for prostate cancer patients with suspected metastasis who are candidates for initial definitive therapy, or with suspected recurrence based on a rising serum prostate-specific antigen (PSA) level. In addition, the use of PSMA PET agents for patient selection for men with metastatic prostate cancer who are candidates for PSMA-targeted radioligand therapy with ^177^Lu-vipivotide tetraxetan (PLUVICTO), is consistent with current PLUVICTO FDA labeling and National Comprehensive Cancer Network (NCCN) guidelines 21,22 (Table 1).

**Figure 2:**
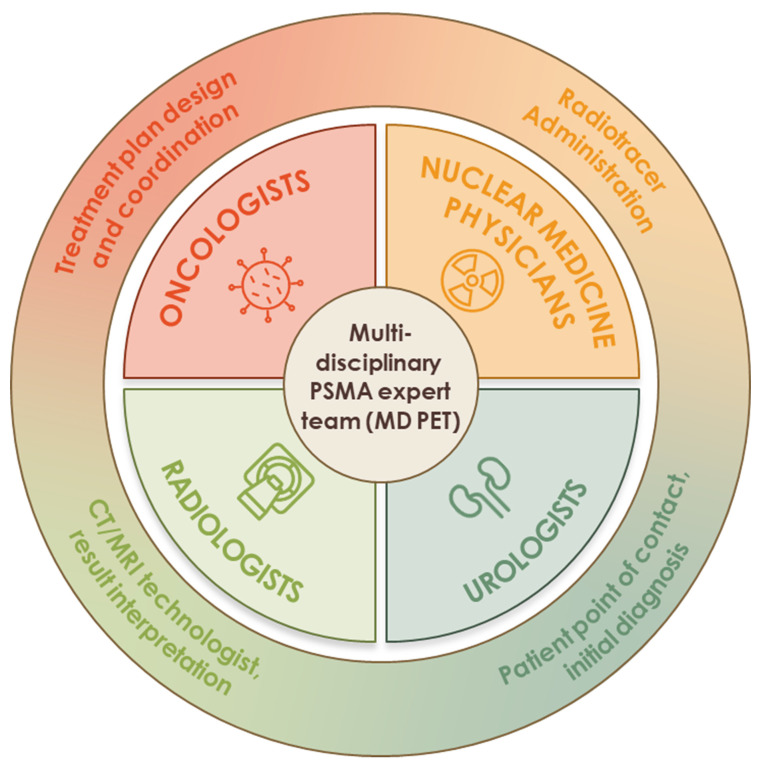
** The multi-disciplinary PSMA expert team (MD PET).** The multi-disciplinary PSMA expert team (MD PET) consists of 4 core disciplines: oncology, urology, nuclear medicine, and radiology. Multidisciplinary care may improve patient-specific decision-making.

PSMA PET agents have significantly changed the diagnostic landscape of prostate cancer by enabling more accurate staging, restaging, and treatment planning 23,24. This article, which brings together a Multi-Disciplinary PSMA Expert Team (MD PET) working group (Figure 2), provides a unique perspective that highlights the unmet need and knowledge gaps in the clinical application of PSMA PET in prostate cancer and summarizes ongoing trials aimed at addressing these gaps. It also provides strategies to overcome current challenges while exploring new opportunities to enhance the clinical utility of PSMA PET.

## Access Barriers to PSMA PET Uptake and Use: Overview of the Unmet Need

### Infrastructure barriers

The availability of imaging infrastructure significantly influences the utilization of PSMA PET. Fixed, onsite PET scanners that are typically housed within large academic or specialty centers require substantial capital and ongoing operational investment 25 and limited numbers and locations contribute to long wait times. As a result, access remains limited across many community healthcare settings.

### Coverage barriers

Coverage is another key barrier to PSMA PET access, influenced by both the patient population and the policies of individual insurance providers. For example, among Veteran Affairs (VA) populations, access to PSMA PET appears to be less restricted with a small percentage of scans ordered off-label for low- and favorable intermediate-risk prostate cancer cases 26. In contrast, non-VA populations can face more challenges due to variability in payer policies 27. Moreover, the choice of PSMA PET agent is often determined by the insurance provider 28. Though the NCCN guidelines support the use of any FDA-approved PSMA PET agent for patient selection prior to ^177^Lu-PSMA-617 therapy, factors such as pricing beyond the initial three-year coverage period may influence agent choice in clinical practice. These payer-driven constraints may limit optimal agent selection based on patient-specific clinical scenarios. Furthermore, the issues of insurance and reimbursement barriers are not limited to the US. In both Europe and Asia, due to its high cost, PSMA PET is not always covered thus requiring patients to bear the out-of-pocket cost of imaging related expenses or lose access altogether 29,30.

### Addressing this Unmet Need

#### Infrastructure expansion through funding and access programs

Investment in PET infrastructure, including both fixed and mobile units 31, may be facilitated through public-private partnerships, federal funding initiatives, or value-based healthcare models 32. Expanded access programs, such as those sponsored by the Department of Veterans Affairs or National Cancer Institute-designated cancer centers, may improve equity in imaging access across both urban and rural settings 33.

Accreditation programs offered by professional societies such as the Society of Nuclear Medicine and Molecular Imaging (SNMMI) and the American College of Radiology promote standardized acquisition protocols for PSMA PET/CT 23. Facilities accredited under these programs ensure high-quality imaging and minimize scan variability across sites.

#### Insurance reform

Payer-driven restrictions may delay diagnosis or limit patient access to necessary care 27,28. Reimbursement policy changes are needed to minimize coverage-related variability across providers and improve accessibility to PSMA PET. Advocacy from clinical societies and real-world data demonstrating clinical utility plays a crucial role in mediating meaningful changes in the policy.

#### Cost-effectiveness evidence

Beyond infrastructure expansion and insurance reform, studies have been conducted to determine whether the use of PSMA PET for prostate cancer is cost-effective. An analysis performed by Privé et al. reported that for PI-RADS 3 lesions, PSMA-PET/CT was marginally cost-effective, improving per-patient Quality-Adjusted Life Years (QALY) by 0.002 34. Separately, Yee et al. reported that PSMA PET with piflufolastat F 18 was a cost-effective option for men with prostate cancer in the US, with higher associated life-years, QALY, and greater net monetary benefit than non-PSMA-targeted imaging agents 35. While more pharmacoeconomic and outcomes-based studies are needed, preliminary evidence suggests that PSMA PET can be cost effective for some prostate cancer patients.

## Inconsistent Understanding on When to Order a PSMA PET Scan: Overview of the Unmet Need

Although there is an increase in the adoption of PSMA PET, clinician ordering patterns vary considerably in terms of timing and indication for scans 36. This could be due to evolving guidelines, limited imaging experience, or the biological heterogeneity of the disease itself. Such inconsistency in PET ordering practices could lead to either over or underutilization of the PSMA PET, potentially affecting disease staging and treatment planning. Multidisciplinary care involving urologists, radiation oncologists, medical oncologists, and nuclear medicine colleagues, when available, may improve patient-specific decision-making 37.

### Addressing this unmet need

Clinical guidelines have emphasized the appropriate integration of PSMA PET in prostate cancer staging and treatment planning 22-24. The 2026 NCCN guidelines recommend the use of PSMA imaging in the initial staging of patients with unfavorable intermediate- and high-risk prostate cancer as well as in patients with biochemical recurrence following curative-intent therapy 22. Additionally, PSMA PET is recommended for patients with metastatic castration-resistant prostate cancer (mCRPC) when assessing eligibility for PSMA-targeted radioligand therapy. The NCCN recognizes the superior sensitivity and specificity of PSMA PET tracers compared to conventional imaging modalities, such as CT, MRI, and bone scan, in detecting disease at both initial staging and biochemical recurrence. The panel further notes that conventional imaging is not a necessary prerequisite for PSMA PET and that PSMA PET/CT or PSMA PET/MRI can serve as an equally effective front-line imaging tool 22. Although ^68^Ga-PSMA-11 is the only agent formally approved for selecting patients for ^177^Lu-PSMA-617 radioligand therapy, the NCCN panel acknowledges that piflufolastat F 18 and flotufolastat F 18 may also be used based on their comparable imaging performance 22. Furthermore the U.S. prescribing information for ^177^Lu-PSMA-617 for patients with PSMA-positive mCRPC prior to chemotherapy is now agnostic to the choice of PSMA PET agent for patient selection only requiring that the PET product be FDA approved 21.

## Knowledge Gaps When Conducting and Interpreting PSMA PET: Overview of the Unmet Need

### Variability in scan performance across sites

The performance of PSMA PET scans varies across different clinical settings (e.g., academic institutions, freestanding outpatient centers, etc.) due to differences in scanner technology, accreditation status, reader expertise, as well as variability in imaging protocols and imaged field of view among FDA-approved PSMA PET agents. PET/CT scanners vary in design with differences in detector technology, sensitivity, resolution, and reconstruction algorithms impacting overall performance 38-41. Studies comparing digital PET to conventional analog PET have demonstrated variations in standardized uptake value (SUV) measurements and improved detection of small lesions with digital platforms 40,41.

Reader variability also contributes to inconsistencies in image interpretation. Interobserver differences in PSMA expression scoring and lesion classification are well documented and influenced by readers' training and experience 42,43. Nuanced clinical knowledge of different disease states and their associated imaging requirements (e.g., initial staging, biochemical recurrence [BCR], mCRPC) is crucial for accurately interpreting PSMA PET scans in various scenarios. While academic centers may possess experienced readers, the equipment may be antiquated in some facilities. In some clinical trials, sites are ineligible to participate if the PET scanner is older than 10 years; however, such cutoffs do not exist in real-world settings. Therefore, some PET centers may continue to use outdated scanners. Conversely, freestanding outpatient centers are often less likely to be accredited and may utilize less advanced scanners. These centers tend to get reimbursed less and often shorten the imaging protocols to improve patient throughput. Additionally, technical factors such as the choice of radiotracer, urinary bladder volume, and acquisition timing can influence image quality 23,44-46.

### Inadequate reporting

Radiology reports often lack the level of detail necessary for effective treatment planning. Reports that fail to specify lesion location or omit image references may leave referring clinicians with insufficient information for decision-making 47. Additionally, fused images stored in picture archiving and communication systems (PACS) are often difficult to interpret. Without access to advanced software many clinicians are unable to adjust the images to locate described lesions or confirm findings, limiting the clinical utility of the scan. Furthermore, imaging metrics such as whole-body SUV_mean_ have been shown to predict outcomes in the VISION and TheraP trials 48,49. However, no standard reporting or nuclear medicine reports present such information. Incorporating such metrics into routine reporting could enhance clinical decision-making and is possible with artificial intelligence (AI) integration.

### Shortage of trained nuclear medicine physicians

There is a shortage of trained nuclear medicine physicians in the United States 50. While radiologists are permitted to interpret scans, they are not board-certified in nuclear medicine, and interpreting complex scans requires extensive experience. The number of Accreditation Council for Graduate Medical Education (ACGME)-certified nuclear medicine training has declined significantly over the years while demand has increased, widening the gap 50. This shortage of trained experts also presents a challenge for implementing AI, as it is difficult to account for all the variables involved in scan interpretation 51.

### Addressing this unmet need

#### Optimizing performance and reading of scans

The optimal image quality depends on standardized scan acquisition protocols. Factors, such as the injection process, acquisition time, and bladder volume, influence image quality 22,52. Since different tracers may exhibit distinct uptake and biodistribution patterns, the choice of radiotracer directly affects scan performance 53. Optimizing injection protocols, including timing, dosage, and administration, enhances lesion detectability while minimizing artifacts 23,54. While the typical imaging time for a PSMA PET scan is 60 minutes post-injection, delayed imaging has shown potential for detecting additional metastatic sites, particularly in cases of small-volume disease 23,55,56. Longer acquisition time improves image quality by increasing detectability and reducing noise 46.

Urinary bladder volume is also an important factor, as high tracer accumulation can create artifacts that blur images and obscure pelvic lesions 23,45. This interference is especially relevant for PSMA-ligands with kidney-dominant excretion, where residual activity in the urinary system may lead to false positive or negative findings. Strategies such as pre-scan hyperhydration, bladder voiding, and use of diuretics are commonly recommended to reduce urinary tracer interference and improve scan quality 23. Additionally, selecting tracers with lower urinary excretion may further minimize bladder-related artifacts 45,57. However, well controlled, prospective head-to-head comparative studies are needed to validate whether this approach improves diagnostic accuracy.

Multicenter studies require standardized imaging protocols to ensure reproducibility across institutions. Without direct head-to-head trials comparing different imaging agents, interpreting scans with varying tracers remains a challenge.

#### How to avoid interpretive pitfalls

With the challenges of false positives and tracer-related variability, there are a number of approaches to mitigate these potential issues. First, having a familiarity with typical lesions unrelated to prostate cancer can help with the interpretation of false positives. For instance, benign findings such as granulomatous prostatitis in the prostate, fibrous dysplasia and Paget's disease in bone, and desmoid tumors in soft tissue can be properly contextualized to avoid misinterpretation. Another example is modest PSMA uptake in the inguinal lymph nodes which is a common observation but typically does not suggest pathologic involvement. Isolated metastatic rib lesions are also often difficult to distinguish from a healing fracture, and it is well known that other tumor types can appear on PSMA PET. Lastly, standardized reporting systems such as PSMA-RADS may help to address issues related to reader variability 58.

#### Enhancing report utility

Imaging reports play a crucial role in guiding treatment decisions 23,47. The utility of the report can be enhanced by providing the target site and image slice to help guide the reader. Community clinicians may benefit from reports that in addition to summarizing the findings, provide educational insights into technical aspects as well as guidance on scan interpretation. Standardized reporting of PSMA PET can improve communication between imaging specialists and referring physicians, ensuring that key findings are conveyed clearly and consistently 47.

#### Potential of AI to support interpretation

AI-driven tools can enhance reader ability by providing quantification of key prognostic and predictive metrics, such as total tumor volume and whole-body SUV_mean_ 59. The aPROMISE platform is designed to standardize tumor burden evaluation with piflufolastat F 18 PET/CT and represents a significant advancement in prostate cancer imaging 60. Compared to conventional imaging, aPROMISE-assisted reads significantly upstaged patients with localized and regional prostate tumors and reduced inter-reader variability 60. Johnsson et al. reported that aPROMISE effectively automates anatomic contextualization, lesion detection, and SUV-based quantification, thereby supporting standardized reporting of PSMA PET/CT in clinical practice 61. Recently, a study has reported high agreement between automated (aPROMISE) and semi-automatic manual segmentation methods in identifying dominant and index tumors 62. Integrating AI-driven tools, such as aPROMISE, into the PSMA PET workflow can improve standardized reporting, efficiency, and diagnostic accuracy. However, larger prospective studies are required to validate its performance and support clinical adoption 63. AI is not a replacement but a valuable adjunct, particularly in settings with limited access to nuclear medicine specialists.

Furthermore, the AI space is rapidly evolving with advances being made beyond basic anatomical segmentation. PSMA-based AI has been able to assist with theranostic treatment response and differentiate between intraprostatic disease and more advanced grades including extra-prostatic extension. However, there are still a number of obstacles to broader adoption including a lack of standardized protocols, the need for further validation studies, and improved integration into clinical workflows 64,65.

## Adoption of PSMA PET for Staging to Guide Clinical Decision-Making: Overview of the Unmet Need

Despite the growing evidence of PSMA PET for detecting prostate cancer lesions, its adoption is inconsistent across clinical practice 66. An area that lacks robust data is the utilization of serial PSMA PET imaging in assessing patient response/progression and in guiding management 67. The lack of validation studies has hindered broader adoption into routine clinical decision-making. In this context, some treating physicians have limited its use to patient selection and response assessment when using PSMA-directed targeted therapy. Recently, the Response Evaluation Criteria in PSMA PET/CT (RECIP 1.0) has been developed to standardize the assessment of systemic treatment response in metastatic prostate cancer using PSMA PET/CT 68. In addition, the recently published Prostate Cancer Clinical Trials Working Group 4 (PCWG4) guidance recommends new criteria for the use of PSMA PET in validating treatment response and progression in prospective clinical trials 69,70. Given the limited clinical evidence linking PSMA PET findings to improved therapeutic outcomes, some physicians may continue relying on conventional imaging.

Though PSMA PET is increasingly used for patient selection, conventional imaging remains the standard for follow-up response assessment in prostate cancer trials, despite its limitations in detecting lesions 67. A key unmet need is the ability to determine high-and low-volume metastatic hormone-sensitive prostate cancer using PSMA PET, which might help identify patients who could benefit from the addition of docetaxel chemotherapy. In addition, it is not uncommon for patients selected based on PSMA-positive scans to lack corresponding target lesions on conventional imaging, complicating response assessment. As a result, some recent trials (such as ARROW, NCT03939689; 71) have begun evaluating PSMA PET alongside bone and CT scans during therapy assessment. Future studies should directly compare conventional imaging and PSMA PET in a head-to-head design, generating essential conversion data that will facilitate the gradual transition from conventional imaging as more evidence accrues.

### Addressing this unmet need

PSMA PET has significantly influenced the management of prostate cancer across different risk categories. Studies evaluating the clinical utility of PSMA PET have demonstrated changes in staging, treatment planning, and therapeutic strategy 72.

#### Unfavorable intermediate-risk disease

While most clinical trials have focused on high-risk or recurrent disease, recent evidence suggests that PSMA PET may also influence management decisions in patients with unfavorable intermediate-risk prostate cancer. In this group, PSMA PET has revealed nodal involvement or distant metastases that were not detected on conventional imaging, leading to changes in treatment 73. A retrospective multicenter analysis reported a higher positivity rate for nodal and distant metastases in unfavorable intermediate-risk patients compared to favorable intermediate-risk patients, with PSMA PET impacting therapeutic management in 13.3% of cases overall 74. The PSMA-dRT trial, a multicenter phase 3 study, evaluated PSMA PET before definitive radiotherapy in patients with unfavorable intermediate- and high-risk prostate cancer 75. PSMA PET/CT upstaged 17% of patients relative to baseline staging, which resulted in more accurate radiotherapy planning, including radiation volume delineation, dose escalation, and hormone therapy intensification. Unfavorable intermediate-risk prostate cancer is at the crossroads of indolent and aggressive disease and can benefit significantly from the precise staging of PSMA PET and optimizing strategies.

Though PSMA PET is approved for staging in unfavorable intermediate-risk prostate cancer, it is not routinely recommended for favorable intermediate-risk disease 22. However, emerging data suggest that PSMA PET may add value in detecting atypical metastases and false-negative lesions from CT or bone scan in this population 76. These findings highlight a potential overlap between favorable and unfavorable intermediate-risk disease. Future studies are needed to clarify the utility of PSMA PET in favorable intermediate-risk prostate.

#### High-risk disease

In high-risk prostate cancer patients, PSMA PET has demonstrated higher sensitivity over conventional imaging in detecting metastatic disease in pelvic and distant lymph nodes 10,77. Studies have shown that PSMA PET significantly impacts management decisions, leading to changes in radiation planning and systemic therapy initiation or escalation in high-risk prostate cancer patients 75,77,78.

The proPSMA trial, a multicenter randomized study, found that PSMA PET/CT altered management plan in 28% of high-risk patients, influencing strategies such as shifting from curative-intent surgery to systemic therapy 77.

Recently, a study evaluated the usefulness of the PSMA scores in reporting PSMA PET/CT in high-risk prostate cancer patients 79. It reported a change in disease staging in 39% of cases following PSMA PET/CT, and PSMA score leading to a change in therapeutic decision-making in 32% of patients 79. These findings underscore the crucial role of PSMA PET in optimizing treatment strategies and guiding clinical management of high-risk patients.

#### Biochemical recurrence

In patients with BCR following definitive therapy, PSMA PET has revealed previously undetected local recurrences 80, as well as nodal involvement and distant metastases 12,80,81, even at low PSA levels 81. The identification of locoregional versus distant recurrence directly influences salvage strategies, guiding treatment tailored to the disease burden. The CONDOR study, which evaluated piflufolastat F 18 PET in patients with rising PSA and non-informative standard imaging, demonstrated a change in intended management in 63.9% of BCR cases following PSMA PET 81. These changes included transitions from observation to initiating therapy, shifts between systemic and local therapies, and a shift from planned treatment to observation 81. An Australian prospective multicenter study found that PSMA PET altered management intent in 62% of BCR patients, distinguishing locoregional recurrence in 39% of cases from distant metastases in 16% of cases 82. In patients with very early BCR, PSMA PET led to management changes in 42.8% of cases, including salvage radiotherapy in select patients with pelvic oligometastases and initiation of systemic therapy in cases where extra-pelvic disease was detected 83. These findings highlight the role of PSMA PET in accurately localizing recurrence, improving salvage therapy decisions, and preventing empiric treatment that lacks precise disease identification.

Despite the encouraging impact of PSMA PET on clinical decision-making, direct evidence linking its use to improved patient outcomes remains limited. One prospective study demonstrated that PSMA PET findings were highly predictive of 3-year freedom from progression in post-prostatectomy BCR patients undergoing salvage radiotherapy 84. While several studies have shown association between PSMA PET findings and subsequent management changes across different risk categories, definitive improvements in overall survival, progression-free survival, or quality of life have not yet been confirmed in prospective randomized trials. Larger, multicenter, outcome-driven studies are needed to establish the clinical benefit of PSMA PET and guide its broader adoption. Table 2 summarizes selected ongoing clinical trials of PSMA PET-guided therapy in prostate cancer with patient outcome measures.

#### Patient selection and post-treatment assessment

As it relates to patient selection and post-treatment assessment for PSMA-targeted radioligand therapy (RLT), critically important guidelines have just been published by the Prostate Cancer Working Group 4 (PCWG4) 70. Specifically, while provided within the context of prostate cancer clinical trials, the guidance recommends baseline PSMA-PET/CT for pretreatment imaging whenever feasible. This is essential information given the additional recommendation to expand response and progression criteria as a proposed method to test and validate in the clinical trial setting. Observed changes in SUV_max_, SUV_mean_, or SUV-assessed tumor volume will help determine the extent of treatment response not just to RLT but potentially, non-PSMA targeted treatment paradigms.

As well as PCWG4, Gafita et al., have described the use of SUV_max_, SUV_mean_, or SUV-assessed tumor volume for treatment response evaluation 67. PERCIST, first developed for FDG PET/CT using SUV_peak_, has been modified for PSMA PET with mixed results. In addition, PSMA PET progression (PPP) and RECIP 1.0 criteria have been developed to leverage these measurements to evaluate treatment response. PPP criteria include the appearance of 2 or more new distant lesions that are PSMA-positive or a new lesion consistent with clinical or lab findings and histopathology confirmation or corroborative imaging. Alternatively, RECIP 1.0 defines a new lesion as new focal uptake of a PSMA-targeted agent that is higher than surrounding background with a tumor lesion SUV_max_ greater than blood-pool SUV_max_; its accompanying classification system allows for assessments of tumor responses from progressive disease to complete response 67.

To address the challenge of variability in SUV measurements across different scanners, tracers, and protocols, ongoing efforts, such as EANM/European Association of Nuclear Medicine Research Ltd. (EARL) accreditation, and programs by EARL and the SNMMI Clinical Trials Network (CTN), are aimed at making these metrics robust enough for multi-center trials. Quality assurance and control focused on daily calibration measurements and cross-calibration for F 18- and Ga 68-based agents have been previously described as well as PET/CT manufacturer-based guidance 23,85.

Lastly, it will be interesting to see how these emerging PSMA-based criteria will compare with conventional imaging (e.g., RECIST) and other functional modalities such as FDG PET in the context of future clinical studies. In a recent head-to-head comparison of ^18^F-FDG and PSMA PET/CT, both sensitivity and diagnostic accuracy were greater for PSMA at both 1 and 3 hour imaging time points 86.

#### Potential role of PSMA PET-derived metrics in staging and clinical decision-making

SUV_max_, in particular, has emerged as a useful biomarker in prostate cancer imaging and plays an important role in tumor characterization, risk stratification, and treatment decision-making. Several studies have demonstrated a positive correlation between SUV_max_ and clinical parameters such as Gleason score, PSA levels, and tumor aggressiveness 87,88. Higher SUV_max_ values observed in the prostate gland and metastatic lesions are often associated with more aggressive disease and higher-grade tumors.

In diagnostic contexts, SUV_max_ has demonstrated utility in differentiating malignant from benign lesions, guiding targeted biopsies, and selecting candidates for focal therapies or systemic treatment escalation 89,90. A recent study retrospectively analyzed thirty-four ^68^Ga-PSMA-11 PET/CT studies performed during the initial staging and reported a positive correlation between intraprostatic SUV_max_ and the WHO/ISUP grade group of prostate cancer 91. The study revealed that high-risk patients exhibited significantly higher mean SUV_max_ values compared with low-risk patients, reinforcing the role of SUV_max_ in risk stratification.

SUV_max_ may also help identify suspicious lesions warranting targeted biopsy. A pre-biopsy PSMA PET/CT scan may guide the biopsy site, potentially reducing false negative results 89. Furthermore, detection of lesions with markedly elevated SUV_max_ may lead to intensified treatment strategies such as surgery or systemic therapy, while low-uptake foci in the prostate support active surveillance.

Beyond staging, SUV_max_ has been associated with the pathologic upgrading from non-clinically significant to clinically significant prostate cancer between biopsy and surgery 92. A prospective real-world study established and validated the optimal SUV_max_ cutoff value of 5.3 for distinguishing clinically significant prostate cancer from benign prostate disease 93.

Moreover, SUV_max_ is gaining attention for its ability to predict treatment response and clinical prognosis. Recent evidence suggests that baseline SUV_max_ is closely associated with biochemical response and may help in identifying responders to PSMA-targeted radioligand therapy 94. Overall, these findings emphasize the expanding role of SUV_max_ in the personalized management of prostate cancer.

In addition to SUV_max_, other PSMA PET-derived metrics, such as whole-body SUV_mean_ and total tumor volume (TTV), are recognized as important emerging biomarkers 48,95. Baseline whole-body SUV_mean_ has been shown to predict treatment response to ^177^Lu-PSMA-617 therapy in mCRPC 48,49,96, while PSMA-TTV has demonstrated prognostic value for clinical outcomes by providing a comprehensive measure of disease burden 47,94. Together, these PSMA PET metrics can complement each other in guiding treatment decisions.

## Expert Perspectives

**Figure 3:**
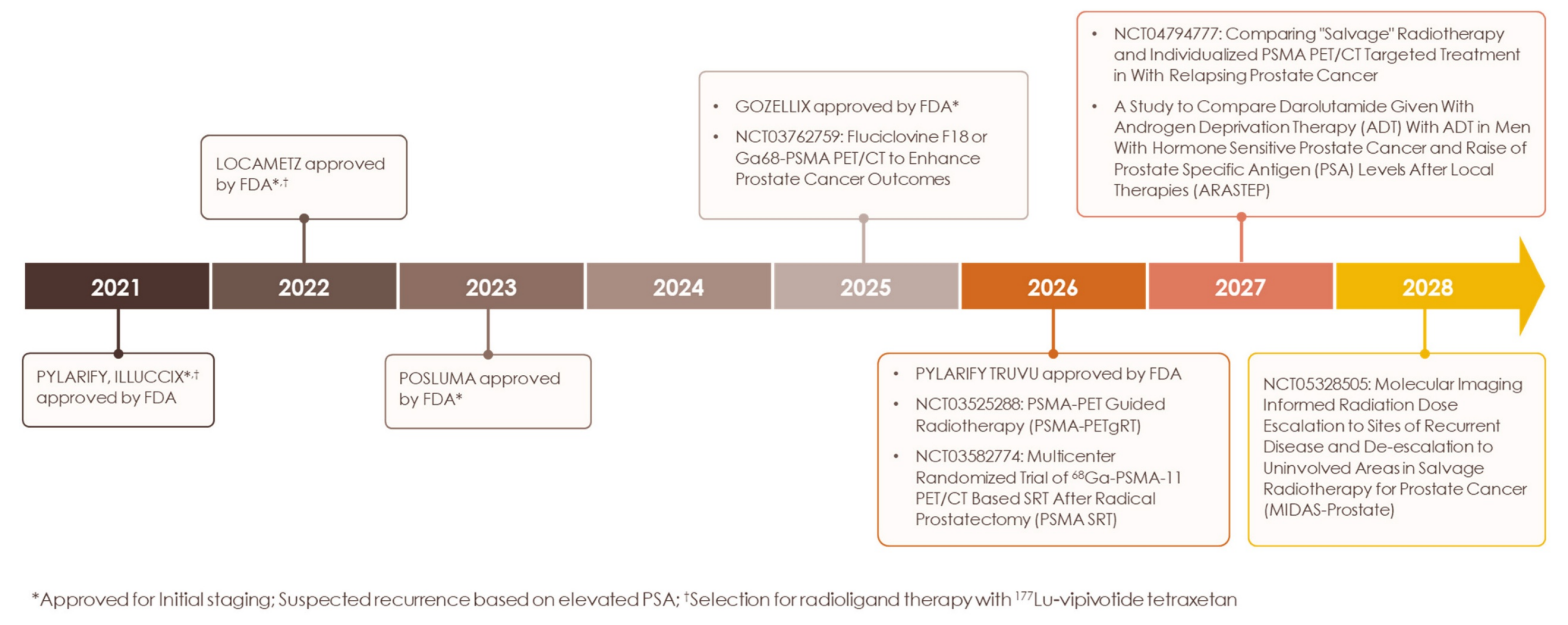
** Timeline of PSMA PET agent milestones.** History of FDA approval for current PSMA PET radiotracers and ongoing clinical trials evaluating current and novel radiotracers in diverse indications.

**Figure 4:**
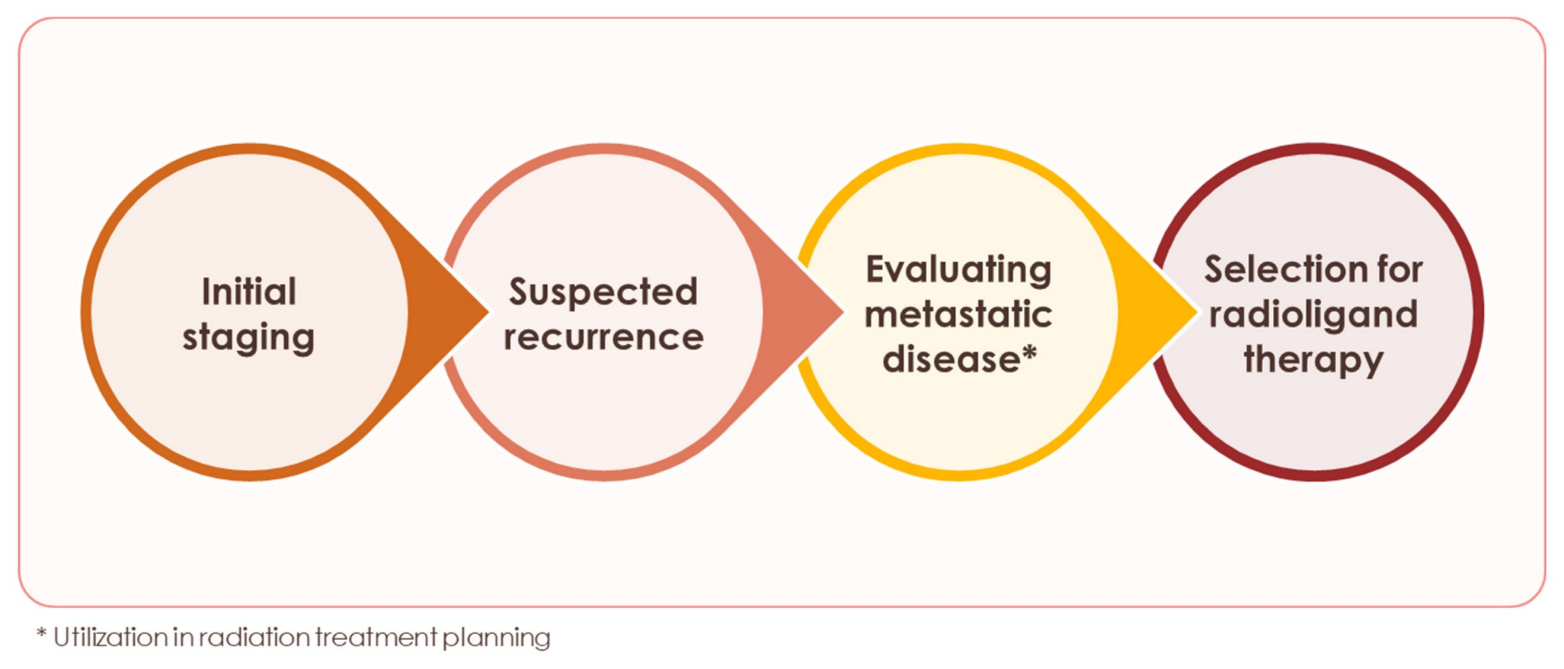
** Applications of PSMA PET for prostate cancer.** Applications where use of PSMA PET is currently recommended.

Starting with the initial FDA approval of Ga 68 PSMA-11 for UCLA/UCSF in December 2020 97,98, PSMA PET has transformed prostate cancer imaging (Figure 3) and, in the process, patient management. We have entered a new era of prostate cancer diagnostics marked by higher accuracy and improved detection of previously unidentified lesions compared to conventional modalities. The recommended applications of PSMA PET continue to expand and include: initial staging, detecting biochemical recurrence, and evaluating metastatic disease (Figure 4) 23. For patients with high-risk prostate cancer, some centers now proceed directly to PSMA PET, bypassing conventional bone or CT scans, a practice supported by updated guidelines. Although PSMA PET is not routinely recommended for low-risk localized prostate cancer, it may be valuable in certain scenarios, such as clarifying inconclusive MRI findings or investigating an unexplained PSA elevation despite negative conventional imaging or biopsy results. This nuanced, case-by-case use underscores the importance of clinical judgment and highlights the need for continued education on evidence-based imaging to avoid both underuse and overuse.

In standard practice, PSMA PET is often used for patient selection and eligibility assessment; however, treatment response is still typically evaluated using conventional imaging, even when these modalities initially fail to detect disease. In clinical trial design, we strongly encourage the use of the same imaging agent throughout the study, from baseline to follow-up, to ensure consistency and enable optimal interpretation.

Different tracers exhibit distinct uptake and distribution patterns; therefore, the choice of imaging agent can significantly influence SUV interpretation. This variability may complicate lesion characterization, particularly in oligometastatic disease. Despite the established role of PSMA PET in prostate cancer, further prospective data are needed to define its utility in intermediate-risk and oligometastatic settings 99,100.

With the publication of PCWG4 70, a consensus framework for incorporating PSMA PET with clinical and PSA criteria, new guidance on its use for treatment response and its integration into clinical trials will help facilitate the integration of meaningful parameters in this critically needed space. These insights will help to provide the necessary context for this cutting-edge guidance.

We also would like to acknowledge advancements in non-imaging biomarker methods. Liquid biopsies are minimally invasive and can offer a complementary, comprehensive systemic approach. The use of serum and urinary biomarkers, such as circulating tumor cells and DNA fragments, offer valuable insights into the molecular composition of tumors. Within this context, the extraction and analysis of exosomes from liquid biopsy samples, such as blood, urine, and semen, have demonstrated significant potential as a source of novel biomarkers for prostate cancer 101.

Lastly, another domain that has gained increasing attention in recent years is the role of PSMA PET in non-prostate malignancies. PSMA is also expressed on endothelial cells within the neovasculature of other solid tumors 102. Emerging evidence demonstrates promising results with PSMA PET in renal cell carcinoma 103, breast cancer 104, glioblastoma 105, and hepatocellular carcinoma 106, suggesting potential avenues for its diagnostic and therapeutic use in non-prostate cancers. This also brings up the importance of addressing PSMA-negative or low-expressing lesions in prostate cancer itself and the need for a future multi-targeted approach that could include promising non-PSMA biomarkers such as cluster of differentiation 46 (CD46), delta-like ligand 3 (DLL3), fibroblast activation protein (FAP), gastrin releasing peptide receptor (GRPR), and human kallikrein 2 (hK2) 107.

## Conclusion

PSMA PET has revolutionized the diagnosis and management of prostate cancer, with its role established in some settings while still evolving in others. Barriers to broader adoption across different clinical settings and among practitioners can be addressed through continued education and research. In the future, integration of AI-based tools or algorithms into routine PSMA PET workflow may enhance efficiency, reproducibility, diagnostic accuracy, and personalized treatment approaches.

## Figures and Tables

**Table 1:** Overview of Commercially Available, FDA-Approved PSMA PET Agents

Product Name	Imaging Agent	Half-Life	Approval Year	Indications & NCCN Guidelines*	Pivotal Studies	Diagnostic Performance Summary
PYLARIFY	Piflufolastat F 18	110 minutes	2021	Initial staging; Suspected recurrence based on elevated PSA; Selection for radioligand therapy with ^177^Lu-vipivotide tetraxetan	CONDOR, OSPREY	CONDOR (BCR) 12,71 CLR: 84.8-87.0% Detection rate: 59-66% OSPREY (Initial Staging - Cohort A) 7,12 Specificity 96% PPV 77% Suspected recurrence/metastases (Cohort B) Sensitivity 95.8% PPV 81.9%
ILLUCCIX	^68^Ga-PSMA-11 (^68^Ga-gozetotide)	68 minutes	2021	Initial staging; Suspected recurrence based on elevated PSA; Selection for radioligand therapy with ^177^Lu-vipivotide tetraxetan	PSMA-PreRP, PSMA-BCR, VISION	PSMA-BCR 14 Detection rate: 54% (95% CI: 50-57%) PSMA-PreRP Sensitivity 47% Specificity 90% VISION: 87% of patients screened met PSMA-positive imaging criteria for therapy selection
LOCAMETZ	^68^Ga-PSMA-11 (^68^Ga-gozetotide)	68 minutes	2022	Initial staging; Suspected recurrence based on elevated PSA; Selection for radioligand therapy with ^177^Lu-vipivotide tetraxetan	PSMA-PreRP, PSMA-BCR, VISION	Same as ILLUCCIX 15
POSLUMA	Flotufolastat F 18	110 minutes	2023	Initial staging; Suspected recurrence based on rising PSA; Selection for radioligand therapy with ^177^Lu-vipivotide tetraxetan	LIGHTHOUSE SPOTLIGHT	LIGHTHOUSE (Initial Staging) 13 Sensitivity: 23-30% Specificity: 93-97% SPOTLIGHT (Suspected Recurrence) Detection rate: 83%
GOZELLIX	^68^Ga-PSMA-11 (^68^Ga-gozetotide)	68 minutes	2025	Initial staging; Suspected recurrence based on elevated PSA; Selection for radioligand therapy with ^177^Lu-vipivotide tetraxetan	PSMA-PreRP, PSMA-BCR	PSMA-BCR 16 Detection rate: 54% (95% CI: 50-57%) PSMA-PreRP Sensitivity 47% Specificity 90%

*As per updated FDA labeling for ^177^Lu-vipivotide tetraxetan and NCCN guidelines, all FDA-approved PSMA PET agents may be used for patient selection. This indication is not included in the original prescribing information for PYLARIFY, POSLUMA, or GOZELLIX.

**Table 2:** Selected Ongoing Trials of PSMA PET-Guided Therapy in Prostate Cancer with Patient Outcome Measures

Trial Name	Clinical Trial ID, Phase & Sponsor	Study focus	Population	Primary Outcomes	Secondary Outcomes	Imaging Agent	Estimated Readout
PSMA-SRT	NCT03582774 Phase 3 Sponsor: Jonsson Comprehensive Cancer Center	PSMA PET/CT-guided salvage RT planning post-prostatectomy	Biochemical recurrence after primary prostatectomy (n=193)	5-year Biochemical PFS	Metastasis-free survival, initiation of additional salvage therapy, change in initial treatment intent	^68^Ga-PSMA-11	Primary completion: July 2026 Study completion: July 2027
PSMA-PETgRT	NCT03525288 Phase 2/3 Sponsor: Centre hospitalier de l'Université de Montréal (CHUM)	PSMA PET-guided intensification of salvage RT	Biochemical recurrence following radical prostatectomy (n=130)	5-year Failure-free survival	Rate of failure, toxicity, survival, QoL, detection yield of PSMA PET	^18^F-DCFPyL	Primary completion: May 2026 Study completion: May 2027
MIDAS-Prostate	NCT05328505 Phase 2 Sponsor: University Health Network, Toronto	PSMA PET-guided dose escalation/de-escalation in salvage RT	Post-prostatectomy BCR with PSMA PET-confirmed locoregional recurrence (n=80)	Grade ≥2 Toxicity for GU	Biochemical failure-free survival, QoL	PSMA PET (unspecified)	Primary completion: August 2028 Study completion: August 2030
EMPIRE-2	NCT03762759 Phase 2 Sponsor: Emory University	Dose escalation using PSMA PET vs ^18^F-fluciclovine PET post-prostatectomy	PSA recurrence following radical prostatectomy with negative conventional imaging (n=140)	2-year Disease-free survival	Decision to offer radiotherapy, decision to treat pelvic nodes, decision to boost between the initial and final treatment decisions, prostate bed clinical target volume (CTV) and planning target volume (PTV)	^68^Ga-PSMA vs. ^18^F-fluciclovine	Primary completion: May 2025 Study completion: December 2025
PSMA recidiv	NCT04794777 Phase 3 Sponsor: Stefan Carlsson	Individualized PSMA PET-guided therapy vs standard salvage RT	BCR post-prostatectomy (n=450)	Primary PSA progression free survival	Time to metastasis, Prostate cancer specific survival, Time to secondary treatment, Differences in quality of life recorded using Patient Reported Outcome Measure (PROM)	^68^Ga-PSMA-11 or ^18^F-PSMA-1007	Primary completion: October 2027 Study completion: October 2027
ARASTEP	NCT05794906 Phase 3 Sponsor: Bayer	PSMA PET-guided systemic therapy (darolutamide + ADT) in high-risk biochemical recurrence	High-risk BCR with PSMA PET-positive lesions, no evidence of metastases on conventional imaging (n=970)	Radiologic PFS by PSMA PET/CT	Metastasis-free survival, time to CRPC, time to SSE, overall survival, QoL	PSMA PET-CT (tracer not specified)	Primary completion: July 2027 Study completion: March 2030

## Abbreviations

MD PET: multi-disciplinary PSMA expert team
PSMA: prostate-specific membrane antigen
CT: computed tomography
MRI: magnetic resonance imaging
PET: positron emission tomography
FDA: food and drug administration
NCCN: national comprehensive cancer network
VA: veteran affairs
SNMMI: society of nuclear medicine and molecular imaging
mCRPC: metastatic castration-resistant prostate cancer
SUV: standardized uptake value
BCR: biochemical recurrence
AI: artificial intelligence
RECIP: response evaluation criteria in PSMA PET/CT
PCWG4: prostate cancer clinical trials working group 4
TTV: total tumor volume
